# Identifying Changes in Selective Constraints: Host Shifts in Influenza

**DOI:** 10.1371/journal.pcbi.1000564

**Published:** 2009-11-13

**Authors:** Asif U. Tamuri, Mario dos Reis, Alan J. Hay, Richard A. Goldstein

**Affiliations:** National Institute for Medical Research, London, United Kingdom; Imperial College London, United Kingdom

## Abstract

The natural reservoir of Influenza A is waterfowl. Normally, waterfowl viruses are not adapted to infect and spread in the human population. Sometimes, through reassortment or through whole host shift events, genetic material from waterfowl viruses is introduced into the human population causing worldwide pandemics. Identifying which mutations allow viruses from avian origin to spread successfully in the human population is of great importance in predicting and controlling influenza pandemics. Here we describe a novel approach to identify such mutations. We use a sitewise non-homogeneous phylogenetic model that explicitly takes into account differences in the equilibrium frequencies of amino acids in different hosts and locations. We identify 172 amino acid sites with strong support and 518 sites with moderate support of different selection constraints in human and avian viruses. The sites that we identify provide an invaluable resource to experimental virologists studying adaptation of avian flu viruses to the human host. Identification of the sequence changes necessary for host shifts would help us predict the pandemic potential of various strains. The method is of broad applicability to investigating changes in selective constraints when the timing of the changes is known.

## Author Summary

Influenza A's natural reservoir is waterfowl. Sometimes avian virus genomic segments are able to shift to a human host, either *in toto* or by combining with those that underwent a previous host shift event. Such host shift events can cause worldwide pandemics in their immunologically naive hosts. In order for these host shifts to establish a stable lineage, the virus has to adapt to the new host. Identifying the changes that have occurred in the past can provide important clues about how this process happens, and how surveillance for new influenza threats should be targeted. Unfortunately, it is difficult to determine whether an amino acid has changed due to adaptation to the new host or whether the change occurred through random drift. Here we describe a novel phylogenetic approach to identifying locations where the nature of the selective pressure exerted on the location has changed corresponding to the host shift event. We identify a set of locations on a number of the genomic segments. The approach we describe is of wide applicability when the timing of the change of selective constraints is known in advance.

## Introduction

Influenza A has the distinction of being an old disease, a recurring disease, and an ‘emerging’ disease. Influenza A viruses are found in humans as well as in other animals including swine, horses, sea mammals, and birds, of which waterfowl are considered the natural reservoir [1]. Subtypes of influenza A are distinguished by two surface glycoproteins; haemagglutinin (HA), the primary target of the immune response, and neuraminidase (NA). There are sixteen known types of haemagglutinin (H1 to H16) and nine of neuraminidase (N1 to N9), all found in waterfowl. Only H1, H2, H3 and N1, N2, however, are known to have caused epidemic disease in humans. The predominant forms of influenza A currently circulating in humans are H1N1 and H3N2.

There are two distinct problems represented by influenza. Firstly, the various subtypes currently in circulation in humans cause significant morbidity and loss of life. Secondly, periodically a subtype of influenza can make the shift from aquatic birds to humans, possibly through an intermediate host, resulting in a widespread pandemic in an immunologically-naïve population. These ‘antigenic shifts’ can occur either through the transfer of an entire virus from one host to another, or through a re-assortment process where genomic segments of the avian virus mix with genomic segments currently circulating in humans. In 1957 three virus segments (HA, NA, and PB1) from an avian-like source were combined with the other five segments already circulating in humans to create the H2N2 ‘Asian flu’ pandemic, while in 1968 two segments (HA and PB1) from an avian-like source were combined with the other six from the already-present human H2N2 virus to form the H3N2 ‘Hong Kong flu’ pandemic [2]. It has been suggested that the 1918 H1N1 ‘Spanish flu’ virus was the result of a single host-shift event from birds to humans [3]–[5] but this remains controversial [6]–[9]. In recent years, a number of different avian subtypes have caused sporadic human infections, including H5N1, H7N3, H7N7, and H9N2 [10]. While there is evidence for sporadic transmissions of these avian viruses between humans, the genetic changes necessary for widespread human-human transmission have, so far, seemingly not occurred.

A number of different proteins have been implicated in determining host ranges. Influenza haemagglutinin binds to sialic acid linked to galactose on the surface of the targeted cell; the differing nature of the sialic acid-galactose linkages in birds and humans (α2,3 sialic acid linkages in the bird gut, α2,6 sialic acid linkages of the upper human respiratory tract [11]) provides an important barrier to host shift events. A number of amino acid substitutions have occurred in human influenza haemagglutinin (e.g. Q226L and G228S in H2 and H3, E190N/D and G225E/D in H1) to adjust to the different receptors [12]–[16]. Neuraminidase, the protein responsible for cleaving the haemagglutinin from the receptor surface, also seems adapted to the particular sialic acid linkages, as well as for the pH and temperature of the host tissues [17]. Proteins in the viral replication complex (PA, PB1, PB2, and NP) have also been implicated in limiting host range by restricting replication and intra-host spread in mammals (for a review, see [18].) Of particular note is the PB2 gene, where one specific substitution, E627K, was identified and characterised experimentally as crucial for replication and intra-host spread in mammals [19]–[21].

As part of the widespread surveillance effort, it is important to understand the process of host shifts, and to identify the important changes that are necessary for the shift to occur, or that make the shift more likely. We currently have many examples of both avian and human viruses, so there have been a number of efforts at identifying ‘genetic signatures’ that characterise the virus as adapted to one or the other host. The most common method is to identify sites where the distribution of amino acids found in the virus in one host are sufficiently different from the distribution of amino acids found in the same site in viruses that affect the other host [22]–[24]. Unfortunately, there are two fundamental problems with this approach.

Firstly, the observed changes could represent the result of neutral drift rather than anything specific to the nature of the different hosts. As the human viruses are more closely related to each other than they are to the avian viruses, it would be expected that there would be characteristic amino acids found in the human lineages that are distinct from those found in the avian lineages because of the ‘founder effect’ [25], that is, the maintenance of the idiosyncratic properties of the particular virus that first infected humans. Comparisons of amino acid frequencies in viruses from the two hosts cannot easily distinguish between those that accidentally accompanied the host shift event and those that were actually associated with different selective constraints acting on the viruses in the two hosts.

**Figure 1:**
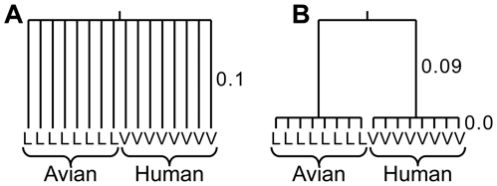
Possible evolutionary scenarios. Two possible phylogenetic trees representing the situation where eight different avian sequences have a L in a given position, while eight different human sequences have a V. (Branch lengths are not drawn to scale.) The situation shown on the right provides much weaker evidence for a shift in selective constraints compared with the situation shown on the left.

The second related problem is the use of inappropriate statistical tests to identify when these two distributions are sufficiently different. The statistical tests used generally assume that each of the observed sequences represent a set of independent measurements. But the underlying phylogenetic relationships will generate correlations in the amino acids at a site, confounding the signal due to the host shift event. This can be demonstrated by considering Figure 1, which shows two possible situations where the avian viruses all have a leucine in a given position where all of the human viruses have a valine in the same position. In example A the results are statistically significant, in that the positions are independent, and it is unlikely that the simultaneous parallel changes in sequence occurred at random in the human viruses but not in the avian viruses. In example B there is much less statistical signal, as only one change of amino acid on the branch connecting the human and avian viruses is needed to explain the multiple observations. By neglecting the underlying phylogenetic structure, a single change of amino acid can be interpreted as a large number of independent events, grossly exaggerating the statistical significance.

A number of the published approaches to this problem suffer from the above problems. For example, both Chen et al. [22] and Miotto et al. [24] employed an information-based approach to identify sites where host-specific amino acids can be identified. Their computations of entropy (a measure of sequence diversity) and mutual information (the dependence of the observed residue distribution on host species) are based on considering every observed sequence as an independent data-point, ignoring correlations between the evolutionarily related sequences. Different distributions in the two hosts can be explained due to the founder effect described above, independent of any role these sites have in host adaptation. That is not to say that their results are incorrect, only that these problems make it impossible to determine their statistical significance. Finkelstein et al [23] looked at sites with a significantly higher degree of conservation in human lineages than avian lineages, and identified 32 markers within the M1, NP, NS, PA, and PB2 genes, 26 of them on the polymerase proteins NP, PA, and PB2. This analysis did not consider the phylogenetic relationships explicitly in their calculation of conservation, choosing instead to base their calculation on the frequency of the different amino acids observed in that site in the different hosts. While they employed strict tests for, for instance, multiple hypothesis testing, it is difficult to determine how much their results were affected by considering only frequencies of amino acids to represent the selective constraints, again ignoring the underlying phylogenetic relationships. It is known, for instance, that such counting methods produce very inaccurate amino acid frequencies compared with phylogenetically-based methods [26], and can not generally identify the rate of substitutions in the tree, but only the range of acceptable amino acids.

As described above, the differences in the distribution of amino acids at a given site between avian and human viruses might represent neutral drift or, more interestingly, a change in the underlying selective pressure applied to the virus by the host. Rather than characterising only the difference in observed amino acid distributions, we can instead look directly for evidence of changes in the selective constraints by modelling the phylogenetics explicitly. These selective constraint changes will result in differences in the substitution process, as mutations that arise in one virus or another will have different probabilities of achieving fixation. Thus, changes in selection constraints will manifest themselves as changes in the observed substitution rates. This also allows rigorous statistical methods, such as the likelihood ratio test, to be used to establish statistical significance.

The selective pressure acting on a site can be positive, negative, or neutral. Positive selection, also called adaptive, or more misleadingly [27] ‘Darwinian’, refers to the acceptance of advantageous mutations; negative, or purifying selection involves the rejection of deleterious mutations. Neutral selection pressure involves the chance acceptance of mutations that do not have a significant effect on the fitness. Both positive and negative selection pressure represent strong constraints on the amino acids at a given site; the difference is that during purifying selection the current amino acids generally fulfil these constraints so change is restricted, while during adaptive evolution the current amino acids are not well suited, generally due to changes in the constraints or a selective advantage for diversification, enhancing the rate of evolution until more appropriate residues are found.

Changes in the selective constraints can result in changes in the rate of substitutions at that location. If the initial amino acids do not match the current requirements of that site, there may be an adaptive burst of faster substitutions until the constraints are satisfied. Modifications of the stringency of the constraints, causing a given site to be more or less restricted, may cause a longer-term change in the substitution rate without necessarily causing an adaptive burst. Previous phylogenetic methods have generally focused on identifying changes in the absolute substitution rate [28]–[35] or ratio of non-synonymous to synonymous changes [36]–[38]. The latter method was used, for instance, to identify twelve sites on the influenza A nucleoprotein that seem to have undergone a change in selective constraints corresponding to the switch from avian to human host [39]. While these approaches are often useful, transient position-specific adaptive bursts are difficult to identify given the short duration of the effect. Sites can also undergo shifts in selective constraints without adaptive bursts or detectible changes in substitution rates, especially if the constraints in the two hosts overlap. Monitoring changes in the *nature* of the selective constraints has been much less common [40] and has not been applied to host shift events.

In this paper we investigate the use of a phylogenetic method to detect changes in selective constraints that considers not only changes in the magnitude of selection constraints, but also changes in its nature, represented as the relative propensity for the different amino acids. We do this by considering two different models for each site, a homogeneous model where the selective constraints are independent of host, the other a non-homogeneous model where the selective constraints depend upon the host. The likelihood ratio test can then determine the level of statistical support for rejecting the null hypothesis of no such dependence.

## Results

We start our analysis with a set of human and avian influenza viral sequences and the associated phylogenetic trees for each influenza gene. We consider the different haemagglutinin and neuraminidase serotypes (e.g. H1, H2, H3, N1, N2) separately. For each non-conserved site, we apply increasingly complicated substitution models, using the Likelihood Ratio Test (LRT) to evaluate the statistical support for each further complication.

The substitution models are defined by a symmetric exchangeability matrix **S**, the equilibrium frequencies of the twenty amino acids **π**, and a rate scaling parameter ν representing the relative substitution rate at that site compared with other sites. The simplest model, Model 1, consists of the WAG exchangeability matrix combined with the associated equilibrium frequencies for the different amino acids [41], with one adjustable parameter per site representing the scaling factor ν. We then consider Model 2 where the equilibrium frequencies of the amino acids are optimised individually for each site [26]. The likelihood ratio test demonstrated that the use of site-specific equilibrium frequencies was justified for all sites (*P* values ranging from 0.028 to 9.4×10^−27^).

**Figure 2:**
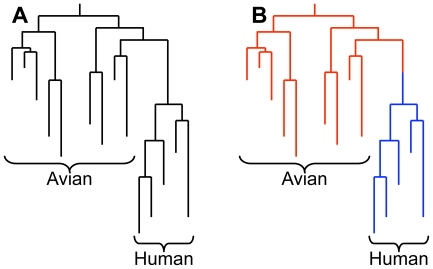
Homogeneous and non-homogeneous substitution models. Illustrative phylogenetic trees showing set of avian and human influenza sequences. A: In the homogeneous models (Models 1 and 2), the same substitution rates are used throughout the tree. B: In the non-homogeneous models (Models 3 and 4) different substitution rates are used for the avian (red) and human (blue) lineages. The root of the tree is assumed to be inside the avian lineage. (Because the model is reversible within the avian clade, the exact location of the root within this clade does not affect the calculation.) The host shift event is assumed to occur at the midpoint of the branch connecting the common ancestor of the human strains with its parent.

We then created a non-homogeneous model, Model 3 where virus substitutions are modelled by one set of substitution rates in the avian host, and by a different set of substitution rates in the human host, as illustrated in Figure 2. The two different substitution models shared the WAG exchangeability matrix **S** and a site-specific rate-scaling factor ν, but now the equilibrium amino acid frequencies were both host- and site-specific. We identified sites with statistical support for different substitution rates in the two hosts, using a false discovery rate (FDR) method to account for multiple hypothesis testing [42]. We identified 172 sites with an FDR<0.05 (*i.e.* we would expect 5% of these sites to be false positives), and 518 sites with an FDR<0.20. We will refer to the 172 higher-confidence locations as ‘A sites’ and the remaining 346 lower-confidence locations as ‘B sites’.

We then considered if modelling differences in the equilibrium amino acid frequencies was adequate, or whether we should include host-dependent rate scaling factors as well. We implemented a more complicated model (Model 4) where the substitution rates were still defined with the WAG exchangeability matrix, but now both the equilibrium frequencies and the scaling factor ν were host- and site-dependent. Of the 2143 sites considered, few (37) had *P* values less than 0.05; after correcting for multiple hypothesis testing using the false discovery rate method, no site yielded any statistically significant improvement. The results described below will be based on Model 3 above.

The list of 172 ‘A’ sites (FDR<0.05) is shown in Table 1. Sites were found on all of the genes considered. Supporting Table S1 shows the list of the 518 ‘A’ and ‘B’ sites with FDR<0.20. Sites that have been identified experimentally are detected using this method, notably PB2 627. HA sites H1 190 and 225 and H3 228 are also identified. Sites H2 226 and 228 are significant at the weaker FDR<0.20 level, while H3 226 is not statistically significant.

**Table 1:** Sites identified as undergoing changes in selective pressure during host shifts from birds to humans.

Location	P	d	Avian residues	Human residues	Avian selective constraint strength (d)	Human selective constraint strength (d)	Cal-4
**H1**							
−5	3.05E-04	2.59E-03	E	K	2.74	2.85	K
2	5.09E-05	7.86E-04	F	L	3.30	2.63	L
7	1.58E-03	0.010	V((A))	A	2.59	2.91	T
8	4.47E-03	0.021	L	T	2.63	2.71	A
15	9.36E-03	0.038	V((I))	I	2.68	2.63	I
54	4.90E-05	7.86E-04	N	K	2.79	2.89	R
63	5.80E-03	0.025	K	N	2.89	2.79	K
70	5.19E-03	0.023	L	I((V))	2.63	2.57	I
77	4.58E-03	0.021	D	E(G)	3.07	2.25	E
80	1.86E-03	0.011	T	S((P))	2.73	2.26	T
91	2.57E-03	0.014	S(T)	P	2.06	3.13	P
120	7.69E-04	5.68E-03	K	R	2.86	2.82	R
138	0.011	0.042	A	S((A))	2.91	2.24	A
141	1.55E-04	1.48E-03	Y	H	3.50	4.01	H
154	4.62E-05	7.86E-04	I((L))	L	2.51	2.63	L
155	1.01E-05	7.42E-04	T(I)	T	2.20	2.73	V
159	1.57E-04	1.48E-03	N(T)	G((S))	2.41	2.49	N
160	0.011	0.042	S	L((S))	2.50	2.41	S
163	1.89E-04	1.70E-03	K	N((T,S))	2.89	2.52	K
187	3.47E-03	0.017	T((N))	N((S))	2.61	2.71	T
188	4.16E-05	7.86E-04	T((V,A))	I((S,T,M))	2.23	2.20	S
189	3.84E-04	2.96E-03	S(G)((D,N))	G(K,R)((T,E,D))	1.64	1.36	A
190	1.76E-09	2.99E-07	E	D(V)((N))	2.74	2.20	D
192	0.012	0.044	Q	(K,M,R)	3.34	1.98	Q
193	4.10E-03	0.020	N(E)((T,S))	(A,T,N)	1.80	1.84	S
197	5.62E-03	0.025	N	T(K)	2.79	2.15	N
198	3.23E-04	2.62E-03	T((V,A))	E((G,V))	2.28	2.47	A
214	0.011	0.042	T((N))	T	2.49	2.70	K
222	2.46E-03	0.013	K((R))	K	2.62	2.89	K
225	6.46E-05	9.15E-04	G	D((G,N))	2.60	2.83	D
238	1.82E-05	7.42E-04	D	E	3.07	2.70	E
239	4.90E-05	7.86E-04	Q	P	3.34	3.21	P
244	1.50E-03	0.010	T	I((M))	2.73	2.56	T
248	2.08E-03	0.012	T	N((S))	2.73	2.67	T
261	1.21E-03	8.54E-03	N	S((N))	2.79	2.45	E
262	1.46E-04	1.48E-03	K	R	2.89	2.82	R
271A	1.55E-04	1.48E-03	D	N	3.07	2.79	D
272	2.98E-03	0.015	A(T,V)	A	1.97	2.87	T
274	2.18E-05	7.42E-04	V((I))	M	2.73	3.42	V
279	0.011	0.042	T	A((S))	2.73	2.77	T
280	1.90E-03	0.011	R(K)	K	2.18	2.89	T
285	1.48E-05	7.42E-04	H((Y,R))	Q	3.60	3.20	K
288	4.90E-05	7.86E-04	L	I	2.63	2.66	I
300	7.61E-05	9.95E-04	I	V	2.66	2.79	I
309	1.76E-03	0.011	V(I)	V	2.49	2.80	V
310	1.26E-04	1.48E-03	K	R	2.89	2.80	K
323	8.95E-03	0.037	V	I	2.83	2.61	I
HA2 72	3.32E-03	0.033	N	K	2.79	2.89	H
HA2 77	2.08E-04	4.17E-03	I	M	2.66	3.47	K
HA2 116	2.07E-04	4.17E-03	R	K	2.83	2.89	K
HA2 127	7.99E-04	0.011	R	K	2.83	2.89	K
**H2**							
186	5.32E-04	0.018	N	I(N)((K))	2.78	1.95	
197	3.62E-04	0.018	N	E(K,N)	2.78	2.01	
205	1.11E-04	0.011	G	S(V)	2.60	1.96	
HA2 45	5.36E-03	0.042	I(V)	F	2.28	3.30	
HA2 130	5.38E-03	0.042	A	V((A))	2.91	2.57	
HA2 169	5.47E-03	0.042	N	K	2.79	2.89	
HA2 180	2.11E-03	0.042	N((S))	S	2.63	2.50	
**H3**							
−7	2.46E-03	0.037	C(Y)	Y	2.92	3.43	
0	5.04E-05	5.73E-03	(G,S,C)	A(T)((S))	1.79	2.13	
4	2.13E-03	0.035	S((P))	P(S)	2.30	2.70	
57	1.15E-03	0.028	K((R))	Q(R)	2.60	2.72	
63	7.32E-04	0.024	D	N	3.07	2.79	
67	1.34E-03	0.028	(I,V,M)	I	1.86	2.60	
92	6.95E-05	5.73E-03	N((S))	K((T,N))	2.49	2.48	
145	1.37E-03	0.028	N(R,S)	K(N)((S))	1.98	2.00	
213	1.17E-04	6.46E-03	I	V	2.66	2.83	
228	5.59E-04	0.023	G	S	2.60	2.50	
244	1.77E-03	0.033	V	L((I))	2.83	2.42	
**N1**							
3	5.96E-03	0.040	P	P((T,S))	3.21	3.04	P
29	7.25E-03	0.043	M(I)	I	2.81	2.64	I
34	4.03E-03	0.031	V((G,I,A))	(I,V,A)	2.36	1.71	I
42	3.43E-03	0.028	(G,N)((S,D))	S((N))	1.63	2.35	N
46	1.96E-03	0.017	(A,P,V,T,S)	T	1.35	2.72	I
47	4.34E-03	0.031	E(G)((D))	G	1.92	2.59	E
52	1.42E-03	0.013	S	R((G,N,K))	2.50	2.51	S
59	2.08E-03	0.018	N((K))	S((N,R))	2.65	2.19	N
67	6.62E-03	0.043	(L,I,V)	V	1.63	2.83	V
74	3.23E-04	6.33E-03	(L,F,S,V)((I))	V	1.28	2.81	F
80	7.93E-04	9.32E-03	V((R,A,M))	(I,V,K)((T,S))	2.21	1.32	V
157	2.11E-04	5.47E-03	T	A	2.71	2.87	T
189	3.08E-08	6.04E-06	S((G))	G	2.40	2.60	N
214	4.73E-06	3.09E-04	D	E(G)	3.07	2.15	D
220	4.22E-04	6.82E-03	R((G))	K(R)	2.57	2.37	R
221	4.52E-04	6.82E-03	N(G)	K	2.41	2.87	N
264	8.09E-04	9.32E-03	(I,A,V)	T	1.71	2.68	V
274	2.80E-05	9.13E-04	Y	(S,F)((Y))	3.50	2.12	Y
288	5.84E-04	8.18E-03	I(V)	V	2.08	2.83	I
289	8.64E-04	9.41E-03	(I,T,M)	M	1.82	3.47	T
309	6.95E-03	0.043	N(D)	N	2.37	2.79	N
311	2.12E-05	8.30E-04	E((D))	D	2.59	3.07	E
329	7.22E-03	0.043	N	K(E)((R))	2.79	2.43	N
339	1.27E-03	0.012	S((L))	T(Y)((N))	2.40	2.11	S
340	1.18E-05	5.80E-04	(L,S,P)((H))	V((A,H,P))	1.55	2.26	S
341	4.13E-04	6.82E-03	N	D	2.76	3.05	N
351	6.82E-04	8.91E-03	F((Y))	Y	3.01	3.49	F
365	1.20E-03	0.012	T(I,P)	N((S,T))	2.05	2.59	I
382	8.60E-08	8.42E-06	E((G,D))	D(N)	2.35	2.30	G
393	4.35E-03	0.031	I	V(I)	2.63	2.30	I
427	4.44E-03	0.031	I	V(I)	2.66	2.46	I
430	2.23E-04	5.47E-03	R((L))	L((Q,R))	2.59	2.30	R
455	2.60E-04	5.66E-03	G(S,D)	N(D)	1.65	2.47	W
**N2**							
41	1.58E-03	0.025	E((G))	E	2.43	2.74	
50	4.12E-03	0.039	V(A)((T,I))	V((A))	1.98	2.69	
51	5.36E-04	0.012	V((M,T))	M	2.39	3.43	
60	5.79E-03	0.047	R(K)	R	2.30	2.83	
62	4.28E-03	0.039	(I,T,M)((V))	I((T))	1.59	2.59	
70	1.82E-04	6.14E-03	S(H,N)	N	1.96	2.78	
72	2.10E-03	0.027	T(I)((V))	T	2.00	2.70	
77	2.05E-03	0.027	(I,K,T)((V,L))	I((K))	1.36	2.32	
81	2.83E-04	8.22E-03	A((V,M,L,I,T))	(P,L,A,T)	1.92	1.62	
83	7.97E-05	3.24E-03	G(E,D)((K))	E	1.61	2.70	
125	7.62E-04	0.014	(G,S,D)	D	1.63	3.07	
126	3.57E-03	0.038	(L,P,T)((H,S))	P((S))	1.68	3.08	
147	7.60E-04	0.014	G	D((N))	2.60	2.94	
155	4.04E-03	0.039	H	Y(H)	3.96	3.19	
192	3.44E-04	8.74E-03	V(I)	V	2.48	2.83	
216	6.64E-03	0.048	(G,V,A)((S))	V(G,S)	1.45	1.92	
283	8.85E-04	0.015	R(Q)	R	2.36	2.83	
286	2.03E-03	0.027	(I,E,N)((D))	G((D))	1.51	2.45	
315	6.05E-03	0.047	G(S,R)((N))	S(R)	1.53	2.14	
328	4.65E-05	2.36E-03	N	K((R))	2.79	2.70	
331	2.49E-03	0.028	(I,R,G,S)	S(R)((N))	1.38	1.86	
338	4.47E-03	0.039	R(K)	L(Q,W)((K,R))	2.34	1.79	
369	5.46E-03	0.046	D	K(E)	3.07	2.18	
378	2.43E-03	0.028	R(K)	K	2.29	2.89	
381	1.46E-05	9.88E-04	G((D,N))	E(D)	2.36	2.42	
384	4.38E-06	4.45E-04	(A,T,I)((V,N,S))	V(I)	1.18	2.06	
386	2.74E-06	4.45E-04	A((P))	P((S))	2.68	3.09	
396	6.80E-03	0.048	V(I)	V	2.21	2.83	
399	6.69E-03	0.048	D	E	3.06	2.74	
**M1**							
115	2.05E-04	0.011	V	I((V))	2.79	2.55	V
137	1.30E-04	0.011	T	A((T))	2.71	2.76	T
174	1.06E-03	0.029	R	K(R)	2.80	2.20	R
231	4.64E-04	0.017	D	(N,D,S)	3.02	1.70	D
**M2**							
10	9.63E-04	0.039	(L,H,P)	P	2.20	3.21	P
93	4.26E-04	0.035	N((Y,I,S))	(S,I,Q)((N))	2.39	1.49	N
**NS1**							
81	5.08E-04	0.029	I(T)((V,M))	M((V))	1.93	3.39	I
84	4.84E-04	0.029	(V,M,G,S)((L,A,I,T))	T(A)((V))	1.17	1.97	V
215	8.84E-06	1.55E-03	(P,S,L)((T,A))	T((P))	1.65	2.60	P
227	6.65E-04	0.029	E((G,K))	R(G)((E))	2.43	2.19	-
**NP**							
77	1.37E-04	8.33E-03	R(K)	K(R)	2.43	2.52	K
101	1.88E-07	3.45E-05	(E,D,N)	G(N)((D))	1.90	1.96	D
102	3.60E-04	0.013	G	G((R))	2.60	2.44	G
131	2.71E-04	0.012	A	A(R)	2.91	2.51	A
136	6.30E-04	0.019	(L,M,I)	I(M)	1.91	2.32	I
283	3.47E-03	0.049	L	P	2.63	3.21	L
305	1.42E-03	0.032	R(K)	K((R))	2.43	2.80	K
335	9.62E-04	0.025	S	S((F))	2.50	2.38	S
353	3.10E-03	0.047	(V,I,L)((A))	(C,S,F,L)((I,V))	1.45	1.48	I
357	1.96E-03	0.036	Q	K((R))	3.29	2.65	K
375	1.26E-04	8.33E-03	(V,D,E,G)((S,N))	G(V)((E))	1.30	1.97	D
425	1.88E-03	0.036	I	I(V)	2.66	2.11	V
472	2.54E-03	0.042	T	T(A)	2.73	2.31	T
**PA**							
356	2.66E-04	0.035	K((R))	R((K))	2.70	2.68	R
552	1.94E-04	0.035	T	S	2.73	2.50	T
**PB1**							
52	4.10E-04	0.032	K((R))	R(K)	2.74	2.17	K
517	3.63E-04	0.032	I((V))	V(I)	2.58	2.06	V
584	7.67E-07	1.81E-04	R((H))	Q(H)	2.73	2.93	Q
**PB2**							
44	6.18E-04	0.023	A(S)	L(S)	2.53	2.15	A
105	9.68E-05	0.013	T(A)((I,M))	V(M)((I))	2.01	2.42	T
199	2.78E-04	0.023	A	S	2.88	2.50	A
475	5.46E-04	0.023	L((M))	M	2.51	3.50	L
493	1.78E-03	0.039	R((K))	K((R))	2.53	2.70	R
569	2.39E-03	0.048	T((A))	A((S))	2.51	2.69	T
613	1.11E-03	0.035	V(A)((I))	T(I,A)	2.33	1.82	V
627	1.20E-03	0.035	E(K)	K	2.20	2.89	E
661	5.91E-04	0.023	A(T)((V))	T((V))	2.28	2.51	A
682	1.48E-03	0.038	G	S(N)	2.60	1.94	G
684	7.63E-05	0.013	A((T))	S(T)	2.69	1.95	S
702	1.58E-03	0.038	K(R)	R	2.42	2.78	K
740	3.83E-04	0.023	D	D(N)	3.03	2.24	D

Legend: Position: Sites in H1, H2, and H3 are identified with respect to their H3 positions. (Locations with negative positions represent an N-terminal insertion in H1 relative to H3. Similarly, location H1 271A represents an insertion between locations 271 and 272.) Numbering refer to HA1 unless specified HA2. Sites in N1 and N2 are identified with respect to their own indices. P: P value using LRT for non-homogeneous model (Model 3) compared with homogeneous model (Model 4). δ: Minimum FDR value required for this site to be identified. This should not be equated with the probability that this identification is a false positive. Residues Identities: Residue identity is shown unadorned if its equilibrium frequency is greater than 0.5, in single parentheses if its equilibrium frequency is between 0.1 and 0.5, and in double parentheses if its equilibrium frequency is between 0.01 and 0.1. Residues with equilibrium frequencies below 0.01 are not listed. Selective (Sel.) constraints represent the strength of the selective pressure in the two different hosts as defined in Equation 5. Cal-4: Identity of the amino acid at this location in a sample of the recent Swine flu outbreak (A/California/04/2009(H1N1)).

To assess the performance of the technique describe here, we simulated each one of the 264 variable sites in the PB2 gene ten times (2,640 simulations in total). All sites were simulated using the same fixed tree topology. The 22 ‘A’ and ‘B’ sites identified as undergoing selective constraint changes (FDR<0.20) were simulated under the non-homogeneous model, using the parameters obtained by optimizing model 3. Similarly, the 242 locations with no evidence for change in selective constraints were simulated under the homogeneous model (model 2). We then applied the analysis described above to identify locations in the synthetic datasets that had undergone changes in selective pressure. On average, we observed that 1.5% of the locations identified with FDR<0.05 were false positives (false positive rate of 0.08%); this increased to 3.6% (false positive rate of 0.2%) for FDR<0.20. This indicates that the FDR values are, at least for PB2, likely conservative. Of the 22 locations modelled with changing selective constraints, 12.9 were identified with FDR<0.05 (false negative rate of 41%), with 16.2 identified with FDR<0.20 (false negative rate of 26%). The 13 ‘A’ sites were identified more consistently, with 10.1 found with FDR<0.05 and 11.0 found with FDR<0.20. This suggests that there remain more locations undergoing changes in selective pressure than are being identified with the procedure described here.

Our approach relies on the prior construction of an appropriate phylogenetic tree. In order to estimate the effect of phylogenetic uncertainty, we repeated the analysis of the PB2 gene segment with ten different phylogenetic trees obtained through non-parametric bootstrapping. The 13 ‘A’ sites were identified on 79% of the bootstrap trees with FDR<0.05 and identified on 90% with FDR<0.20. 85% of the 22 ‘A’ and ‘B’ sites were similarly identified on the bootstrap trees with FDR<0.20. Conversely, the bootstrap trees identified on average 2% (with FDR<0.05) and 6% (with FDR<0.20) of alternative locations that were not identified on the original tree. These might be false positives for the alternative trees, suggesting a similar amount of false positives on the original tree. Some of these locations, however, may be locations with changes in selective constraints, and thus represent false negatives for the original tree; most of these locations would have been so identified with a higher FDR threshold of 0.50, although these points represent only about 12% of the otherwise unidentified locations.

**Figure 3:**
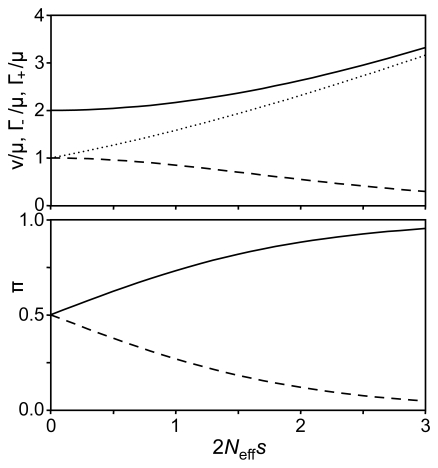
Changing equilibrium frequencies and rates versus selective constraints. Top: Dependence of rate scaling factor ν (solid line) and rate of substitutions for adaptive (positive) selection Γ_+_ (dotted line) and purifying (negative) selection Γ_−_ (dashed line), scaled by mutation rate μ, as a function of scaled selective disadvantage of residue *B* compared with residue *A* (2*N*_eff_ *s*). Bottom: Equilibrium frequencies π*_A_* of *A* (solid line) and π*_B_* of *B* (dashed line) as a function of scaled selective disadvantage.

We constructed a simple model to help explain the lack of statistically significant improvement with adding host-specific scaling factors. This was based on considering a protein site where two amino acids (*A* and *B*) are present, where an organism with residue *B* has a fitness equal to 1−*s* relative to an organism with residue *A*. We used Kimura's fixation rate theory [43] to calculate the resulting substitution rates between *A* to *B*, and formulate these expressions in terms of a rate scaling factor ν and equilibrium frequencies π*_A_* and π*_B_* ( = 1−π*_A_*). We considered how ν, π*_A_* , and π*_B_* change as the relative fitness difference between *A* and *B* is altered. We also considered the overall rate at which substitutions occur in both directions, both for negative selection where the residues are at equilibrium (Γ_−_) as well as for positive selection (Γ_+_) where the location contains the unfavourable residue *B*. Figure 3 shows the dependence of π*_A_*, π*_B_*, ν, Γ_−_, and Γ_+_ (the latter three normalised by the mutation rate μ) on the relative fitness difference *s* (scaled by the effective population size *N_eff_*). As shown, under conditions of negative selection, increasing fitness differences result in a decrease in the overall rate of substitutions, but an *increase* in the rate-scaling factor. There is a relatively weak dependence of ν on *s* as long as the latter is not large relative to 1/*N*_eff_. Under conditions of positive selection, both quantities increase with larger fitness differences.

**Figure 4:**
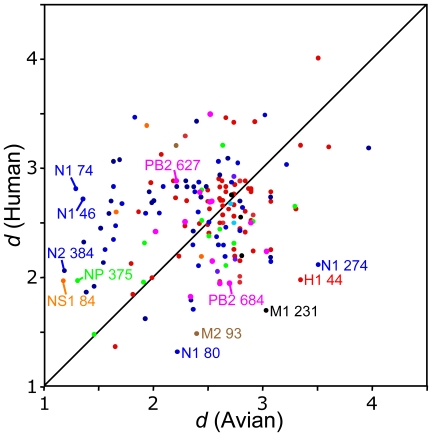
Selective constraint strengths for viral sites in Avian and Human influenza. Strength of selective constraints (measured as *d*, as described in Eqn. 5), for viral sites identified (FDR<0.05) as under different selective constraints in avian and human hosts. Colour coding refers to specific gene: HA (red), NA (blue), M1 (black), M2 (brown), NP (green), NS1 (orange), PA (cyan), PB1 (purple), PB2 (magenta). Selective sites are labelled.

The theoretically predicted weak dependence of ν on selective pressure and the lack of statistical support for host-dependent values of this parameter indicate that ν is not a good measure of the degree of selective constraints. To generate a more appropriate measure, we calculated the relative entropy between the equilibrium frequencies and what would be expected under no selection, **π^0^**, estimating the latter by averaging the amino acid frequencies over our entire database. This measure of selective constraint magnitudes for the various sites in avian and human hosts are presented in Table 1, Supporting Table S1, and in Figure 4.

## Discussion

As described in the introduction, ignoring the underlying phylogenetic relationship often results in a gross over-estimation of statistical significance, as single evolutionary events are interpreted as a large number of independent measurements. Correspondingly, certain sites that have been identified by other methods that do not model the underlying phylogenetics lose their statistical significance when the phylogenetics is considered. For instance, site 271 in PB2 is identified as a significant site in three previous analyses [22]–[24]; human viral sequences are most commonly alanine at this site, while avian viral sequences are predominantly threonine, although alanine also occurs. When each sequence is interpreted as an independent event, there is strong statistical support for host-specific amino acid distributions at this site. All of the alanines in the human lineage, however, can be explained by a single threonine to alanine substitution. In contrast, in the avian influenza there were at least three independent threonine to alanine substitutions. The single example of the substitution in human influenza is not significant given the relative frequency of this transition in avian influenza. Indeed, the more complex Model 3 incorporating host-dependent substitution rates a *P* value of 0.095 compared with Model 2 that assumes no such host-dependence, with an unimpressive false discovery rate of 0.48 after the correction for multiple hypothesis testing. More threonine to alanine substitutions in the human lineage, even if that meant more human sequences with a threonine at this site, would have provided more statistical support. The statistical support would also have been larger if the various avian strains with an alanine at this site represented the result of a single substitution.

The sites that are identified are those with a significant statistical signal given the available data; other sites might be undergoing shifts in selective constraints that are not detected for different reasons. As with all appropriate statistical methods applied to this problem, we require adequate evolutionary time and a suitable substitution rate for the substitution patterns to be detectable. In particular, there has to be sufficient evolutionary time in both the avian and human lineages for the parameters in the substitution models to be sufficiently well defined in each so that the differences in selective constraints are detectible. This will require longer evolutionary time when the selective constraint changes are smaller. As shown in the phylogenetic trees (Supporting Figures S1, S2, S3, S4, S5, S6, S7, S8, S9, S10, and S11), there is relatively little sequence evolution in the human H2 lineage; this is possibly the cause of the relatively few sites identified in this gene subtype. There are more H3 sequences, although most available avian H3 sequences are highly similar, reducing our ability to detect selective pressure changes in this gene subtype. In particular, we do not identify the H3 Q226L mutation whose importance has been determined experimentally, as the strict conservation of glutamine in the avian lineage is not highly informative given the lack of evolutionary divergence among the avian H3 sequences. Finally, the improvement in the log likelihood necessary for a given level of statistical significance is a function in the increase in the number of adjustable parameters between the two models, which is one minus the number of amino acids found in that location. Locations that are highly variable require more adjustable parameters, reducing the power of the likelihood ratio test. In particular, human H3 viruses contain glutamine, leucine, isoleucine, and valine at position 226, making identification of selective constraint changes at this location difficult.

The identified changes in selective constraints may not be the direct result of the host shift event. Selection constraint changes at one site might be a response to substitutions that occur at a different site, even if those changes were themselves the result of neutral drift. We have also assumed that the change in selection constraint occurs simultaneously with the host shift event. In reality this method has limited temporal resolution, and changes in the substitution rate occurring near the host shift event might also be identified.

We do not include ‘pre-selection’ in the model, that is, that the match between the avian sequence and the selective constraints in the human host does not influence the probability that that particular virus strain will undergo a shift to humans. This could be added to such a phylogenetic-based model by considering the probability that a host shift would occur on a given lineage as a function of the protein sequence. This would greatly increase the complexity of the model, increasing the number of adjustable parameters, reducing the statistical power of the method. It is important to note, however, that this would increase the number of false negatives, as these occurrences would look identical to founder effects. It is less likely that this process would produce false positives.

We have included information from the A/California/04/2009 (H1N1) sequences from the 2009 ‘Swine flu’ pandemic in Table 1. This strain represents a reassortment of avian-like European swine genes (M1, M2, NA) with a triple-reassortment strain previously circulating in swine containing segments originally from the classical swine (NP, NS, HA), human (PB1), and avian lineages (PA, PB2) [44]. Considering the locations identified with a false discovery rate of 5%, most segments originally from classical or European swine (NA, M1, NP, NS1) mostly matched the human selective constraints, suggesting a similarity between the constraints in humans and swine. The exception is the HA gene, where many locations seemed to match the avian selective constraints despite its classical-swine origin, possibly reflecting the slow rate of antigenic change of the classical swine haemagglutinin [45],[46]. In the segments more recently from the avian lineages (PA, PB2), most locations more closely matched the avian constraints, while PB2 684 and PA 356 more closely matched the human. Interestingly, by comparing with avian sequences, it appears that PB2 A684S and PA K356R substitutions, both involving changes from an avian-like to a human-like amino acid, occurred in the interval between the host shift to swine and the subsequent transfer to humans, suggesting that these changes might be related to the ability of these viruses to infect humans.

### Changes in equilibrium frequencies versus changes in the rate-scaling factor

Most methods that look for changes in the substitution rates model this as changes in ν, the scaling parameter, or in the related ratio of synonymous to non-synonymous substitutions. In our analysis, we find that, when we allow the equilibrium frequencies **π** to vary, there is no statistically significant variation in ν. This seems initially counter-intuitive, as there are some sites where there seems to be substantial changes in the degree of conservation; in site 274 in N1, for instance, is almost universally tyrosine in avian viruses, while it varies between tyrosine, serine, and phenylalanine in human viruses. Yet the likelihood ratio test applied to this site rejects the inclusion of host-dependent scaling factors with a *P* value of 0.90, suggesting that the relationship between rate scaling factors and site variation are not simply related.

This observation motivated our simple model to try to gain insight into the relationship between equilibrium frequencies and rate scaling factors, by considering a protein site where two different amino acids, *A* and *B*, are found. We imagine that organisms possessing residue *A* at this location have a fitness advantage. Negative purifying selection would occur when the residues at this location are at their equilibrium value, while positive selection would occur when this location was filled by *B*, such as might occur when the selective pressure on the protein changes. By using Kimura's theory of fixation probability [43], we can calculate the values of the rate scaling factor ν, the overall rate of substitutions for purifying (Γ_−_) and positive selection (Γ_+_), and the equilibrium frequencies of *A* (π*_A_*) and *B* (π*_B_*), as a function of the different finesses provided to an organism with the two different possible amino acids at that location, as described in the Methods section below. Normalised values of ν, Γ_−_, and Γ_+_ are plotted as a function of 2*N*_eff_ *s* in Figure 3. As shown, ν varies surprisingly little with *s* as long as *s* is not much more than 1/*N*_eff_. This explains why including a host-specific ν never yielded statistically significant improvements with our data. When we consider adaptive substitutions, larger values of *s* correspond to higher selective constraints, larger values of ν, and faster evolution. The situation is quite different with purifying selection. As might be expected, larger values of *s* (corresponding to larger degree of purifying selection) result in a slower substitution rate, but this actually corresponds to *larger* values of ν. The reason why most phylogenetic programs use an inverted relationship, where larger values of ν correspond to faster substitution rates, is that they do not consider the value of **π** appropriate for each site. By assuming that the same values of **π** apply to all sites, a more extreme distribution of equilibrium frequencies, resulting in a decrease in the number of substitutions, is interpreted as a reduction in ν although this parameter is, in fact, increasing

The magnitude of the selective constraints for the various sites in avian and human hosts are presented in Table 1, Supporting Table S1, and in Figure 4. It is interesting to note the number of positions under changing selection constraints where the magnitudes of the selection constraints are relatively constant. Such sites would be difficult to detect by looking for changes in the substitution rate, especially in cases where the distributions of amino acids found in the two hosts have significant overlap.

The methods described here are applicable for a wide range of problems involving changes in selective constraints. There are two particular factors, however, that make the technique especially well suited for influenza. Firstly, the branch along which the selective pressure changes can be identified *a priori*. Secondly, it is important to generate appropriate phylogenetic trees for the position under consideration. Generation of such trees can be complicated when there is incongruence between different locations. For influenza, incongruence between the various genomic segments results from the process of reassortment, where chimeric viruses containing genomic segments of different origin result from multiple infections. We are able to address this issue by considering each different genomic segment independently, constructing gene-specific phylogenetic trees. A more difficult problem is intra-gene homologous recombination, where different regions of a single genomic segment have different phylogenies. Such recombination is either extremely rare or non-existent in influenza (as well as other negative RNA viruses), and has never been observed experimentally [47]–[49].

We have assumed that the transitions from avian to human hosts did not go through an intermediate species, such as swine. There is no evidence of involvement of swine in the 1957 Asian flu and 1968 Hong Kong flu host shift events. Based on his analysis of the 1918 Spanish flu sequences and the relative timing of the 1918 influenza outbreaks in swine and humans, Taubenberger concluded that the Spanish flu transferred *in toto* from birds to humans and from humans to swine [3]–[5], although this conclusion has been challenged [6]–[9]. If an intermediate host species were involved, it would not be expected to affect the results if the selective constraints at any location in this intermediate host were to resemble either that of avian or human viruses, as this would only change the timing of the shift from one selective constraint to another. If there were an intermediate host and the selective constraints at some locations in this intermediate host were strong and substantially different from either avian or human viruses, the amount of evolutionary time in this intermediate host were sufficiently long, and the evolutionary time in humans sufficiently short so that the new equilibrium is not attained, the results of these calculations could be affected.

There are two other important assumptions made in this work. Firstly, we assume that the selective constraints in human and avian viruses are constant, and that each location can be considered independently. We do not consider, for instance, that there may be different selective constraints in low-pathogenic and high-pathogenic avian viruses, or that compensatory changes can occur elsewhere in the protein or even in other proteins. The observation (both here and experimentally [12]–[16]) that different hemagglutinin subtypes undergo different patterns of change of selective constraints indicates that this assumption is not strictly valid.

## Methods

### Theory

For the following discussion we assume the evolution of a viral protein along a phylogenetic tree with two different host lineages, avian and human, where we consider the root of the tree to exist somewhere in the avian lineage. The evolution of amino acids in a site along a phylogenetic tree can be modelled as a continuous Markov process, described by a 20×20 substitution matrix **Q**. (Standard phylogenetic modelling techniques are described in [50].) In order to provide for time reversibility (that is, the expected number *i* to *j* transitions equalling the expected number of transitions from *j* to *i*), this is commonly represented as 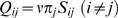 where **S** is a symmetric matrix representing the exchangeability of amino acids *i* and *j*, π*_j_* is the equilibrium frequency of amino acid *j* (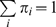) and ν is a scaling parameter that accounts for the overall rate of substitution at the site. **S** encodes the underlying codon structure as well as the relative similarities of the physicochemical properties of the amino acids, while the equilibrium frequencies represent the relative propensities for each of the amino acids at that site. We can calculate the likelihood of the data at this site given the model using Felsenstein's pruning algorithm [51],[52].

We first consider a standard substitution model where **S** and **π** are given by the WAG substitution matrix [41], where each site in the set of proteins is characterised by a distinct substitution rate scaling factor ν whose value is determined by maximising the log likelihood given the sequence data at that site and the input phylogenetic tree. This we refer to as Model 1. We then considered the appropriateness of modelling each site in the set of proteins with a distinctive set of equilibrium amino acid frequencies [26], what we refer to as single-site homogeneous Model 2. We adjust the values of **π** simultaneously with ν to maximise the likelihood. To avoid over parameterisation, we still use WAG **S** values for all sites. The tree topology is assumed fixed, and branch lengths are the same for all sites. In order to reduce the number of adjustable parameters, π*_i_* = 0 for any amino acids not found at that site. As the equilibrium frequencies of the amino acids not observed are set to zero, this results in an increase in the number of parameters equal to the number of amino acids present at that site minus one (due to the constraint that the equilibrium frequencies must sum to one). We then use the likelihood ratio test to see if site-dependent equilibrium frequencies can be justified with the data. As described in the Results section, the site-dependence of the equilibrium frequencies could be justified for all sites.

Now let us imagine that upon inspection of the phylogenetic tree, we notice that amino acid preferences at a particular site seem different in the two host clades. We can incorporate this observation into our model by using two distinct **Q** matrices to describe the evolution of this site in the different hosts, as illustrated in Figure 2. For the reservoir avian host we write 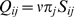 and for the new human host 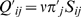 where **π** and **π′** represent the equilibrium amino acid frequencies at that site in avian and human viruses, respectively. (In principle we could also have **S** depend upon the host, but this would result in a large increase in the number of adjustable parameters. We will consider host-dependence of ν below.) The host shift event is defined as the midpoint of the branch connecting the common ancestor of the human viruses with its parent node. We can now calculate a new likelihood for this site using the same fixed topology, again adjusting **π**, **π′**, and ν to maximise the likelihood. We call this the single site non-homogeneous model, Model 3. Again, the increase in the number of adjustable parameters for Model 3 relative to Model 2 equals the number of amino acid types observed at that site minus one. Because the Model 2 is nested inside Model 3, we can again use the likelihood ratio test to test the hypothesis of different selective constraints in different hosts at that site.

In general, for a protein with *N* variable sites, we could repeat the procedure above for each site in the alignment, and perform *N* likelihood ratio tests. This would generate a list of those sites that show statistically different amino acid compositions, and hence distinctive selective constraints, in the different hosts. Following the calculation of the statistical significance for each site we can then use standard false discovery rate (FDR) methods to account for multiple hypothesis testing [42].

Finally, we consider if, in addition to host-dependent equilibrium frequencies, we also have statistical evidence for host-dependent rate scaling factors. We again use 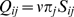 for the reservoir avian host but now use 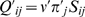 for the new human host where ν and ν′ represent the rate scaling factors at that site in avian and human viruses, respectfully. Again, Model 3 is nested inside Model 4 with an increase of one adjustable parameter, meaning that the statistical support for this extra factor can be evaluated with the likelihood ratio test. We do not observe support for this extra parameter in any of the sites after adjusting for multiple hypothesis testing.

### Data and data analysis

Human and avian viral sequences were collected from the NCBI Influenza Virus Resource [53]. Due to the frequency of reassortment, we cannot assume that the phylogenetic relationships for the various genomic segments are similar; they must be treated independently, including creating genetic-segment specific phylogenetic trees. The sequences for the various segments were treated as independent data sets, with separate datasets for the H1, H2, H3, N1, and N2 genes. Clusters of highly similar sequences (approximately >99.5%) were culled as to reduce the overall number of sequences to around 400 per dataset. It is common to find sporadic transmissions between avian, human, and other (e.g. swine) hosts; we eliminated all sequences resulting from such transmissions (e.g. human H5N1 sequences), leaving us with a single connected set of avian sequences and separate monophyletic human clades corresponding to the host shift events of 1918 (H1, N1, internal genes), 1957 (H2, N2, PB1), and 1968 (H3, PB1).

In order to generate more accurate phylogenetic trees, the culled sequences were aligned at the amino acid level (MUSCLE, [54]), with these alignments then used to create nucleotide codon alignments (PAL2NAL, [55]). The phylogenetic tree topologies were then created for the nucleotide data using PhyML ([56]; HKY85 model [57], Gamma-distributed rates). The resulting trees are included as Supporting Figure S1–S11. Because amino acid distances are needed for the models developed here, branch lengths were then re-optimised for this fixed tree topology using the corresponding amino acid data (PAML [58],[59], WAG substitution matrix [41], Gamma-distributed rates). The analysis was then performed with each gene set, based on the phylogenetic tree for the genomic segment in which the gene is located. A computer program written in Java that implements and optimises the various models described above is available from the authors.

The determinations of changes in selective constraints at each site is a separate hypothesis to be evaluated, so we must address the multiple-hypothesis question, that is, if we ask a suitably large number of statistical questions we are likely, at random, to obtain some statistically-significant results. We use the false discovery rate method, that is, specifying for each site the false positive rate that would have to be tolerated in order for that result to be statistically significant, following the Benjamini and Hochberg estimator [42]. We first choose an acceptable false discovery rate δ. If *P*(*k*) is the *k*-th smallest *P* value for a set of *n* sites, we choose the largest value of *k* so that 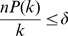. As different genes are evolving in different circumstances, we would not expect the fraction of sites in each gene undergoing changes in selective constraints to be the same. Combining all of the genes together in one dataset would result in an increase in false positives for the genes with fewer changes in selective constraints, and an increase in false negatives for the genes with more changes in selective constraints. For this reason we analyse the false discovery rate for each gene individually. Table 1 and Supporting Table S1 list, for each site, the smallest possible acceptable false discovery rate that would result in that site being labelled as statistically significant. These should not be interpreted as the probability that that given site is a false positive.

### Parametric bootstrapping

Each site was simulated under the homogeneous (Model 2) and non-homogeneous (Model 3) models 10 times using the program *Evolver* [59] using the estimated tree topology and the WAG+F substitution matrix [50]. For each site, the tree was scaled according to the site-specific estimated rate-scaling parameter ν. Simulation under the non-homogeneous model was performed in two steps: the avian part of the tree was simulated using a randomly generated root sequence following the avian equilibrium frequencies for that location. The avian subtree contained a host shift tip that served as the root of the human clade. The human subtree was then simulated according the human equilibrium frequencies using the simulated avian sequence at the host shift.

### Alternative tree topologies

The PB2 sequence was bootstrapped 10 times and tree topology re-estimated for each boot sample. The homogeneous and non-homogeneous models were optimised for the observed data at each location, and the LRT was performed again for each one of the 10 new tree topologies so as to assess the effect of tree topology uncertainty on the identification of adaptive sites.

### Simple model for relationship between equilibrium frequencies and scaling factors

Consider a protein site where two amino acids, *A* and *B*, are found. Let us imagine that that *A* is the more advantageous amino acid, that is, organisms with *A* at this site have a higher fitness, while organisms with *B* at this site has relative fitness 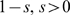. Let us also imagine that the mutation rate from *A* to *B* μ*_AB_* is equal to the reverse mutation rate μ*_BA_* = μ. We imagine a number of different lineages that have diverged, each with effective population size *N*_eff_. Assuming that the mutation rate relative to the population is reasonably small, *A* or *B* will become fixed in each lineage. For haploid organisms, the probability that *A* would become fixed in a given lineage is given by [43]

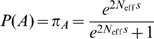
(1)where we have recognised that this probability is simply the equilibrium frequency of *A* in the ensemble of diverged organisms, with 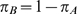.

The substitution rate of *A* by *B* is just the mutation rate *μ* times the fixation probability, given by Kimura's formula for small *s* [43].
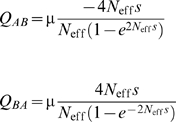
(2)We can compare these expressions with 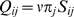 as used in phylogenetic analyses. As we are only dealing with two different residues, 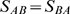 is a simple multiplicative constant and can be set equal to one, resulting in 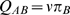. Equating these two expressions for *Q_BA_* and solving for ν yields
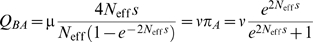
(3)

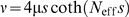
(4)


Similar results are obtained, as would be expected, when we express 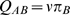.

We can now consider the cases of neutral, adaptive (positive), and purifying (negative) selection. Neutral selection is simply the case when *N*_eff_ *s* is small and 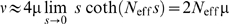. For both neutral and negative selection, we can consider the overall rate at which substitutions occur, given by 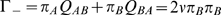, which is equal to *N*_eff_ μ in the case of neutral selection. Positive selection involves the situation where we are not at equilibrium, but rather, at least in this case, we have the less-fit residue occupying the given position. In this case, assuming again that *A* is the favoured residue, 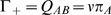.

### Characterising the magnitude of selective constraints

We characterise the selection constraints by how far the equilibrium amino acid frequencies **π** differ from what would be expected under no selection **π^0^** through the relative entropy, defined as
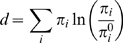
(5)which is, as is desired, zero when **π** equals **π^0^**. Unfortunately, it is difficult to estimate **π^0^**, as there is little of the virus genome that is not under some degree of selective constraints. We estimate **π^0^** by averaging the amino acid frequencies over our entire database, with the expectation that specific selection constraints will, at least approximately, average out.

## Supporting Information

Table S1 Sites identified as undergoing changes in selective pressure with a false discovery rate of 0.20. Position: Sites in H1, H2, and H3 are identified with respect to their H3 positions. Sites in N1 and N2 are identified with respect to their own indices. P: *P* value using LRT for non-homogeneous model (Model 2) compared with homogeneous model (Model 3). delta: Minimum FDR value required for this site to be identified. This should not be equated with the probability that this identification is a false positive. Residues Identities: Residue identity is shown unadorned if its equilibrium frequency is greater than 0.5, in single parentheses if its equilibrium frequency is between 0.1 and 0.5, and in double parentheses if its equilibrium frequency is between 0.01 and 0.1. Residues with equilibrium frequencies below 0.01 are not listed. Selective constraints represent the strength of the selective pressure in the two different hosts as defined in Equation 5 in the text. Highlighted locations are significant with a false discovery rate of 0.05.
(0.11 MB XLS)

Figure S1 Phylogenetic tree of HA genomic segment for subtype H1. Avian section of the tree is in black, human in red.
(2.81 MB PDF)

Figure S2 Phylogenetic tree of HA genomic segment for subtype H2. Avian section of the tree is in black, human in red.
(0.71 MB PDF)

Figure S3 Phylogenetic tree of HA genomic segment for subtype H3. Avian section of the tree is in black, human in red.
(2.94 MB PDF)

Figure S4 Phylogenetic tree of NA genomic segment for subtype N1. Avian section of the tree is in black, human in red.
(3.72 MB PDF)

Figure S5 Phylogenetic tree of NA genomic segment for subtype N2. Avian section of the tree is in black, human in red.
(3.29 MB PDF)

Figure S6 Phylogenetic tree of MP genomic segment. Avian section of the tree is in black, human in red.
(3.02 MB PDF)

Figure S7 Phylogenetic tree of NS genomic segment. Avian section of the tree is in black, human in red.
(2.94 MB PDF)

Figure S8 Phylogenetic tree of NP genomic segment. Avian section of the tree is in black, human in red.
(3.28 MB PDF)

Figure S9 Phylogenetic tree of PA genomic segment. Avian section of the tree is in black, human in red.
(3.25 MB PDF)

Figure S10 Phylogenetic tree of PB1 genomic segment. Avian section of the tree is in black, human in red.
(3.03 MB PDF)

Figure S11 Phylogenetic tree of PB2 genomic segment. Avian section of the tree is in black, human in red.
(3.21 MB PDF)
