# Supplementary figures and images for: Identifying Changes in Selective Constraints: Host Shifts in Influenza
Source: PLoS Comput Biol. 2009 Nov 13;5(11):e1000564. doi: 10.1371/journal.pcbi.1000564 (PMC2770840; doi:10.1371/journal.pcbi.1000564)

# H1

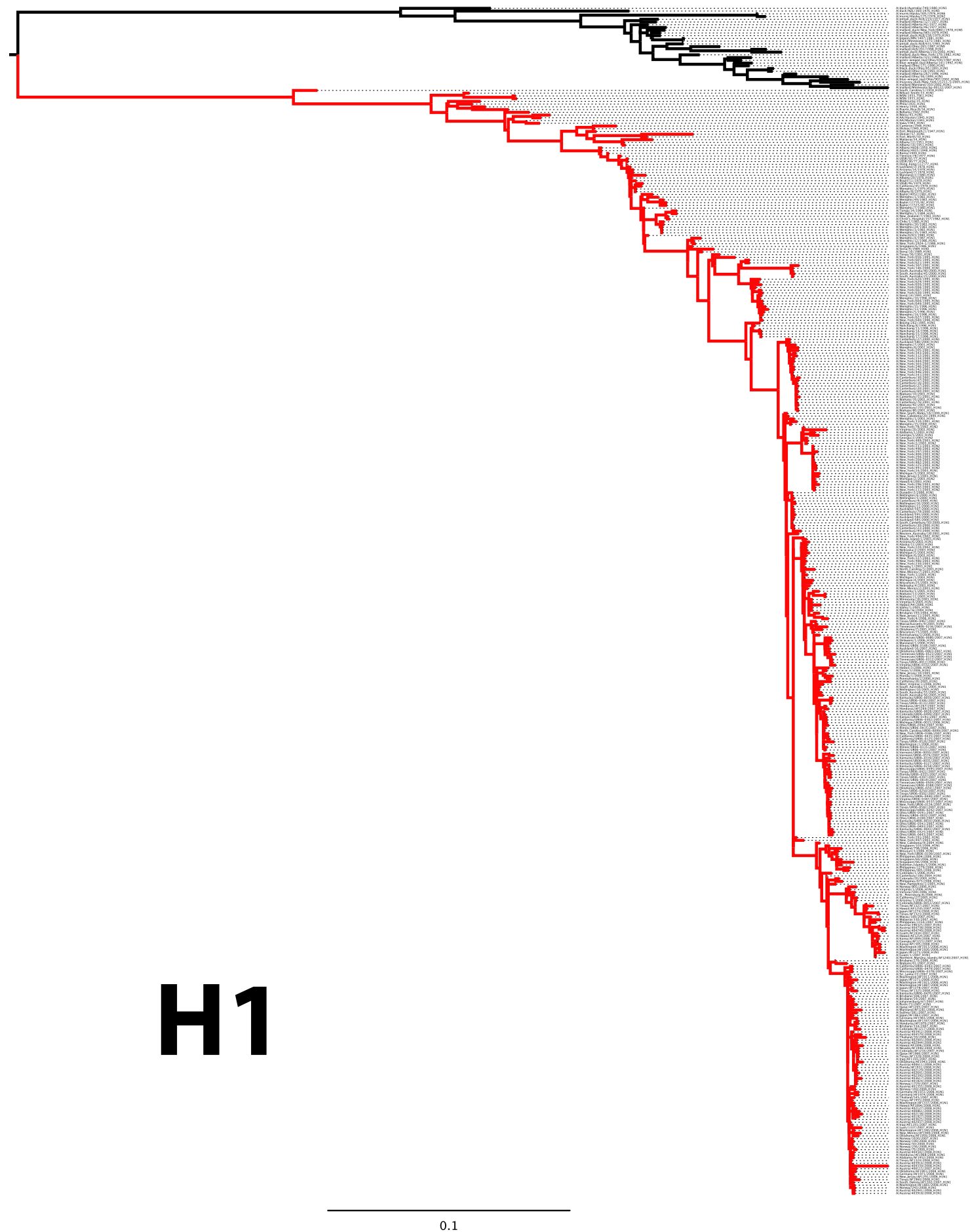

Supplement: Figure S1 — Phylogenetic tree of HA genomic segment for subtype H1. Avian section of the tree is in black, human in red. (2.81 MB PDF) [file pcbi.1000564.s002.pdf]

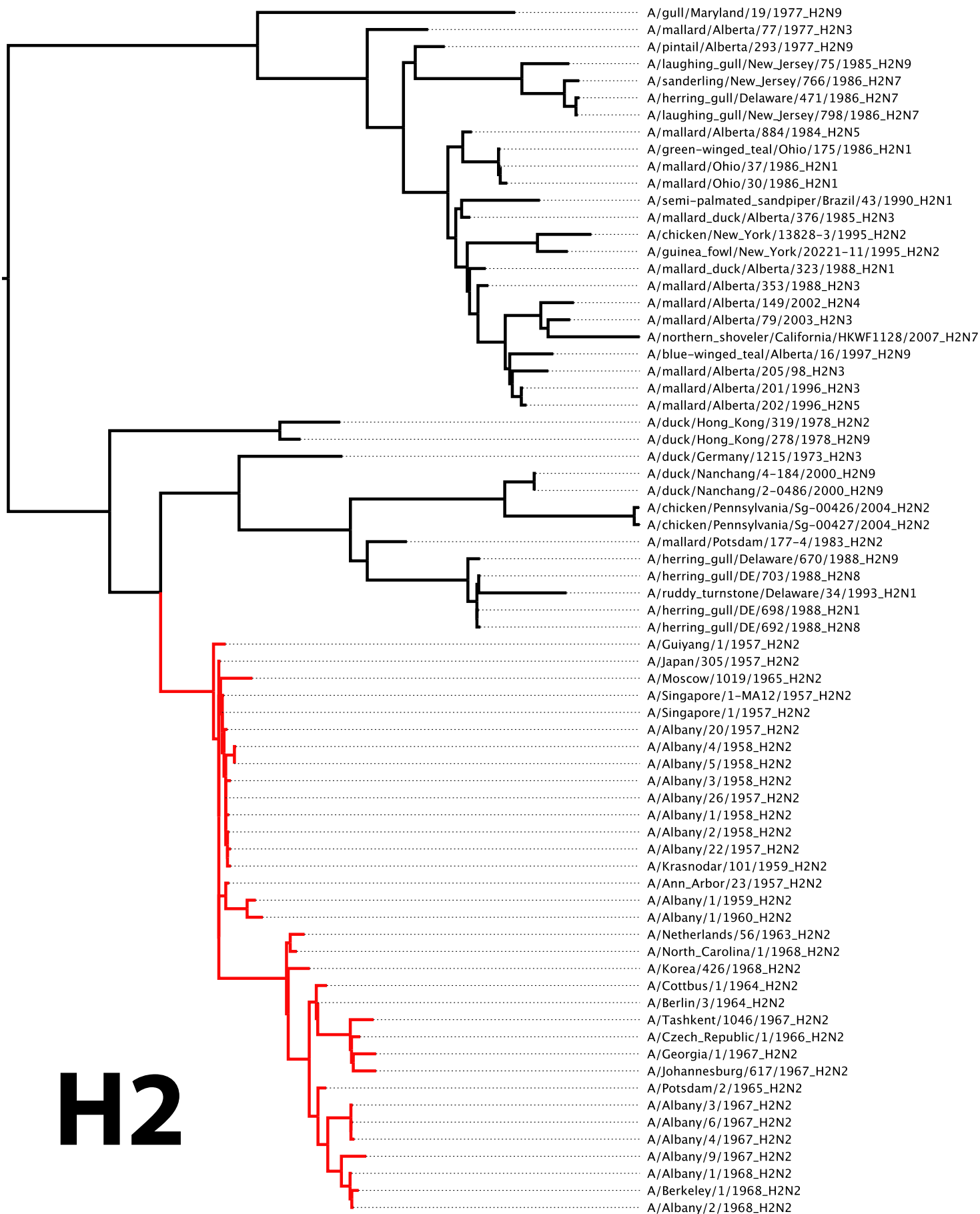

H2

0.1

Supplement: Figure S2 — Phylogenetic tree of HA genomic segment for subtype H2. Avian section of the tree is in black, human in red. (0.71 MB PDF) [file pcbi.1000564.s003.pdf]

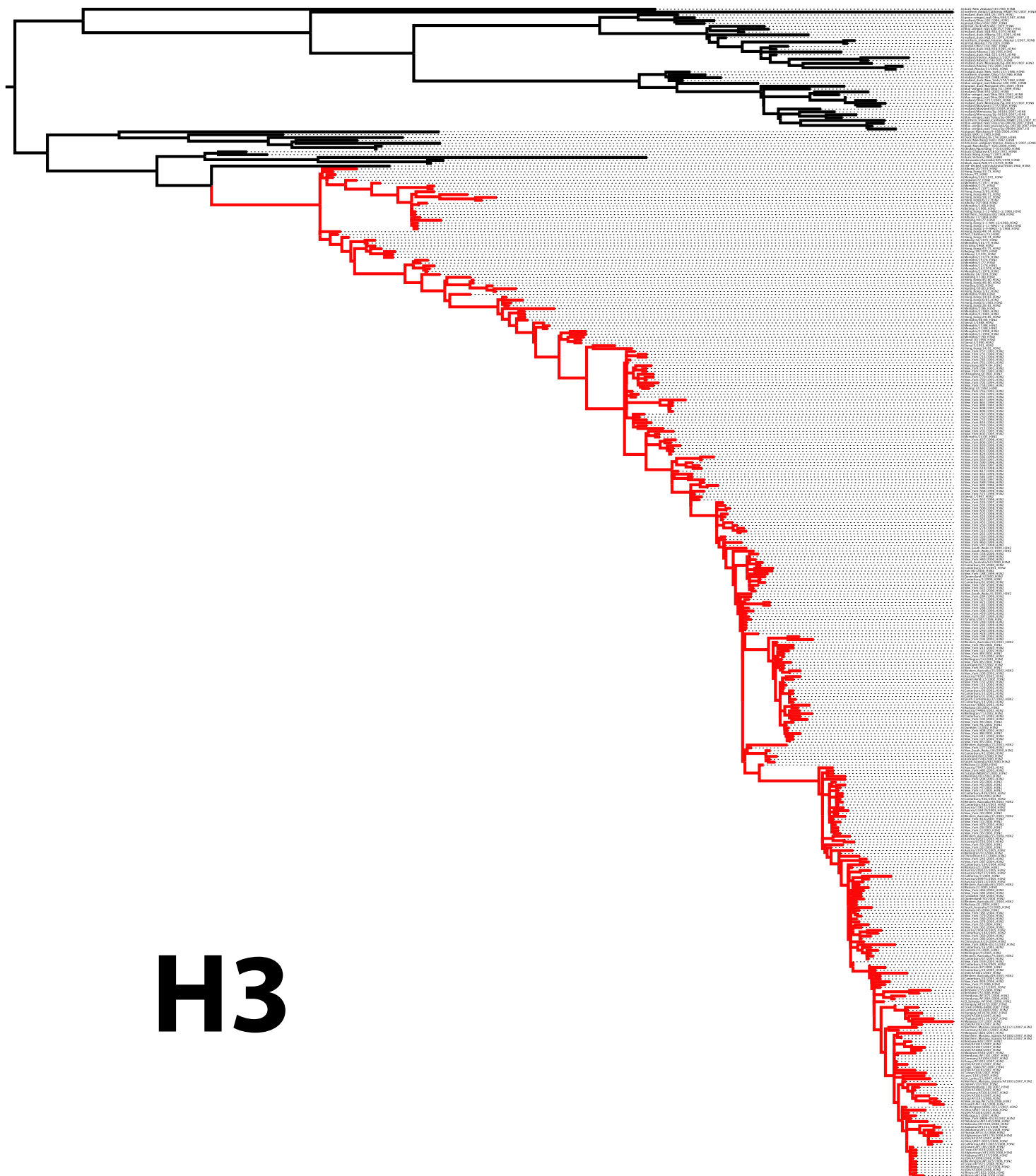

H3

0.1

Supplement: Figure S3 — Phylogenetic tree of HA genomic segment for subtype H3. Avian section of the tree is in black, human in red. (2.94 MB PDF) [file pcbi.1000564.s004.pdf]

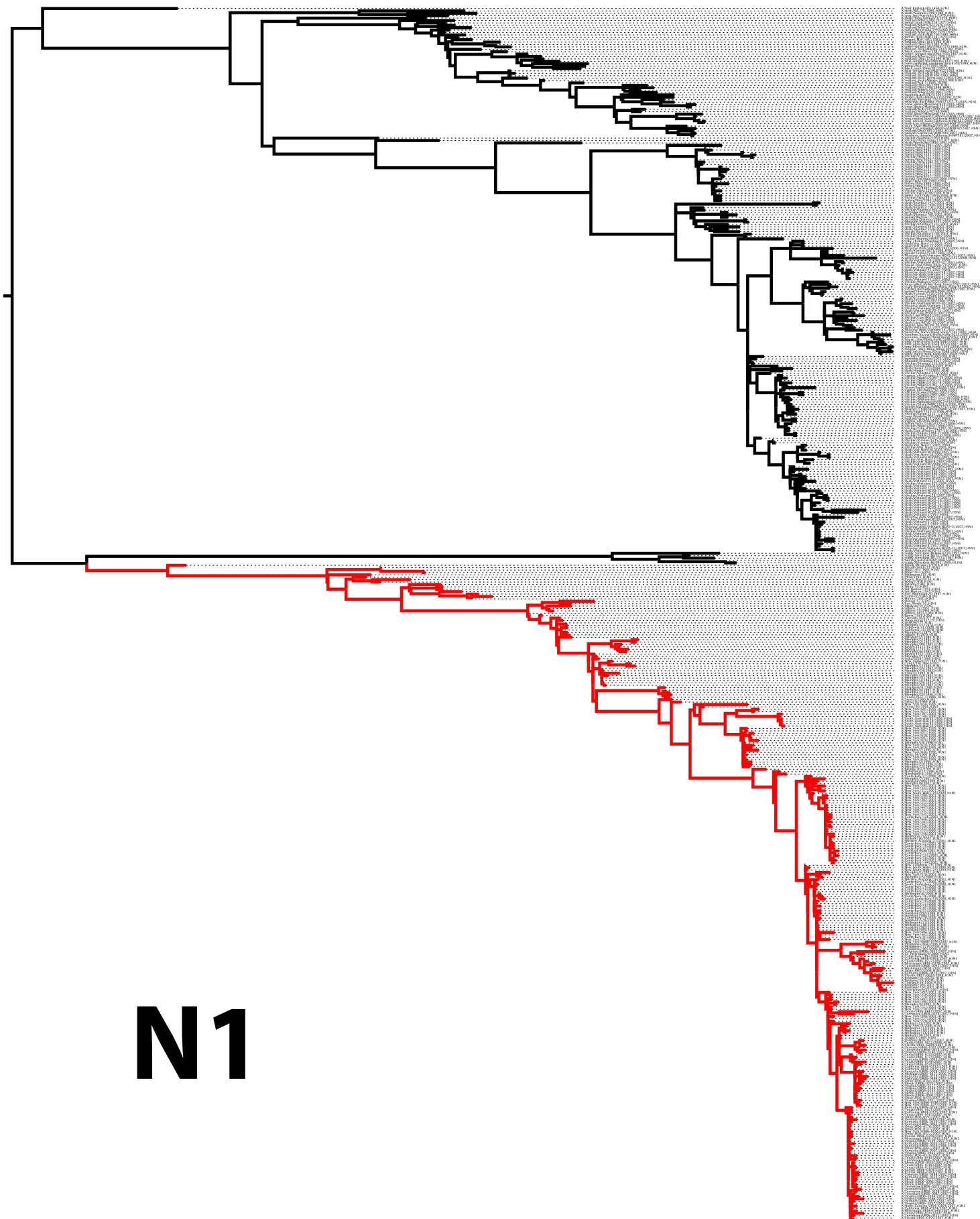

0.1

Supplement: Figure S4 — Phylogenetic tree of NA genomic segment for subtype N1. Avian section of the tree is in black, human in red. (3.72 MB PDF) [file pcbi.1000564.s005.pdf]

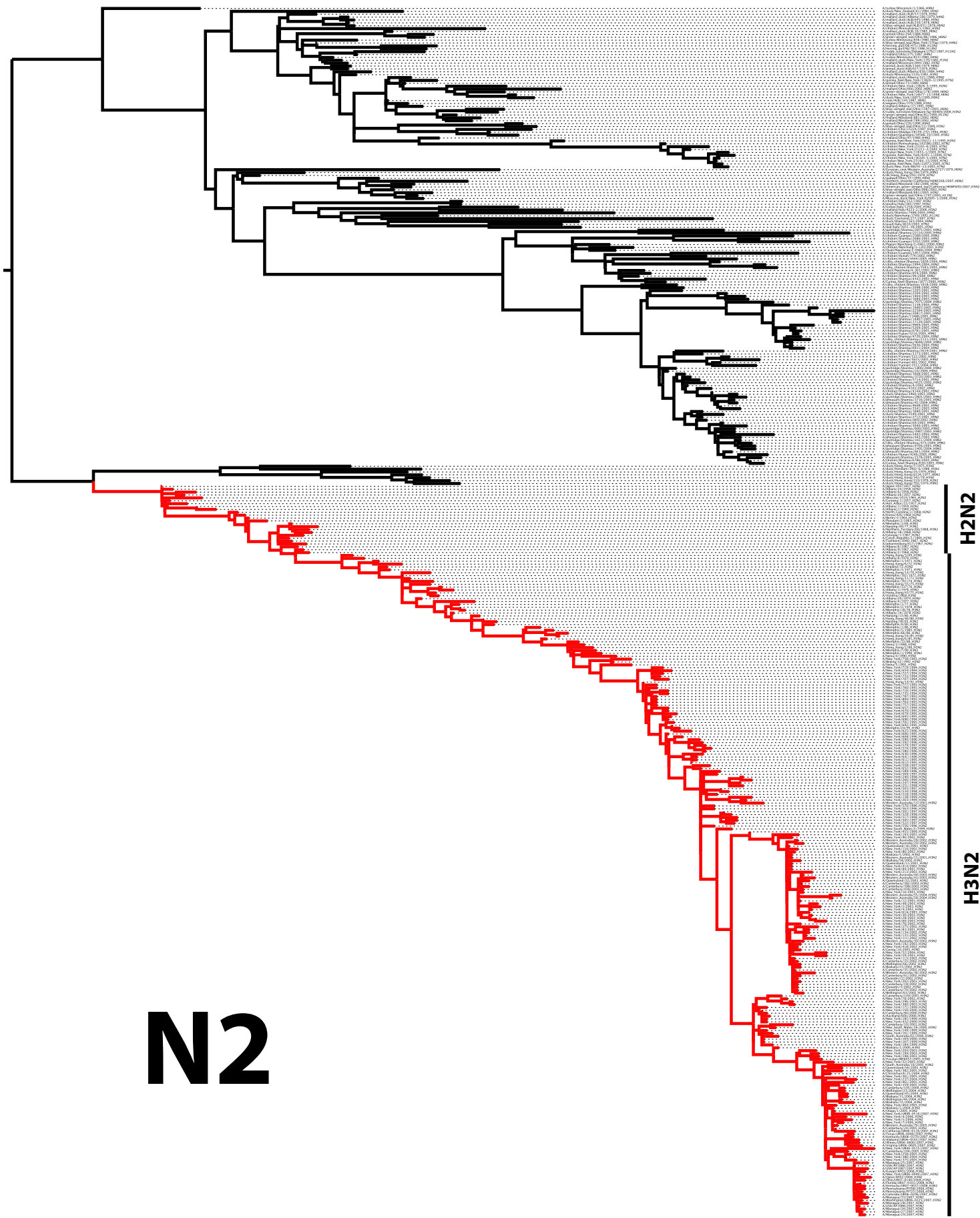

Supplement: Figure S5 — Phylogenetic tree of NA genomic segment for subtype N2. Avian section of the tree is in black, human in red. (3.29 MB PDF) [file pcbi.1000564.s006.pdf]

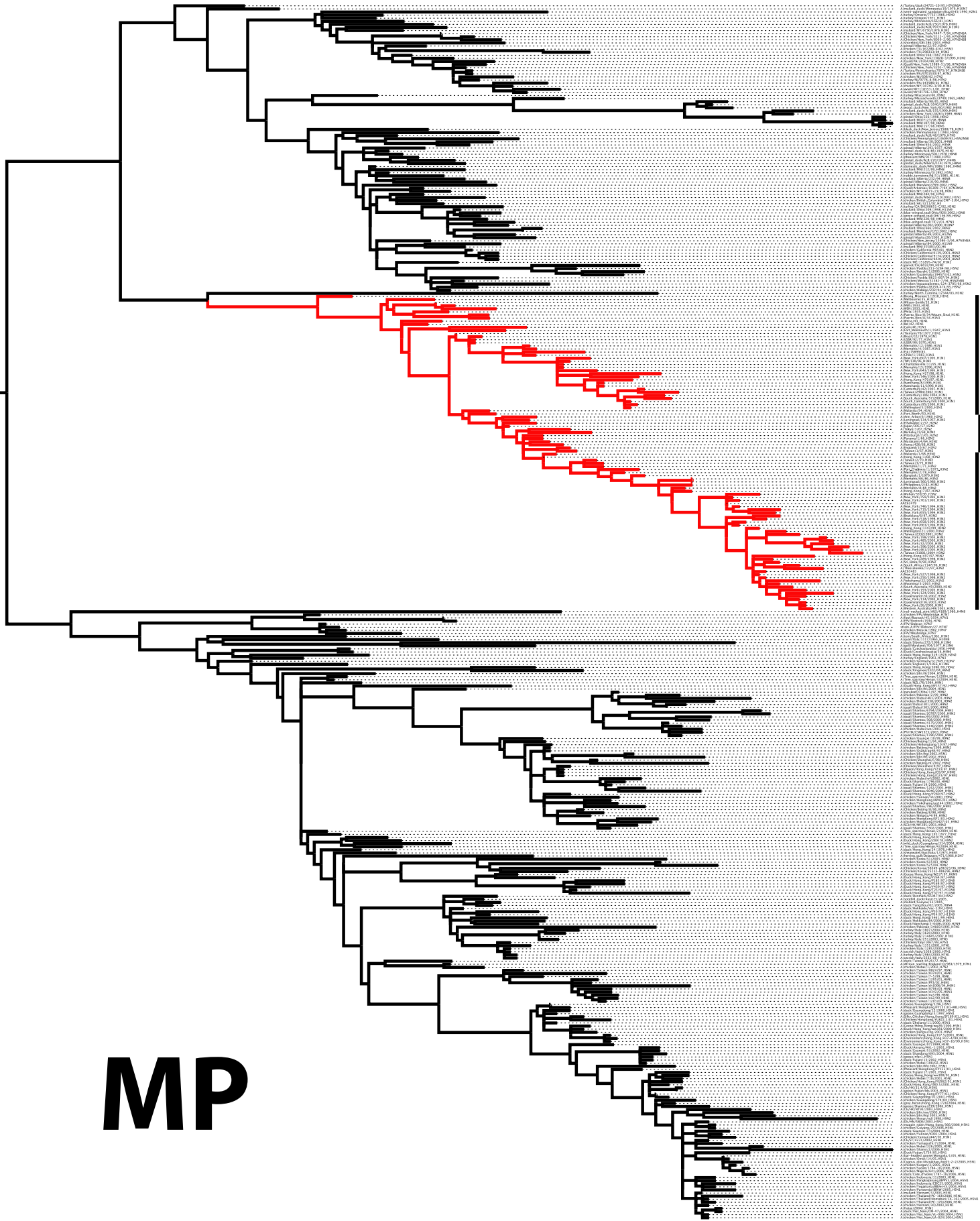

Supplement: Figure S6 — Phylogenetic tree of MP genomic segment. Avian section of the tree is in black, human in red. (3.02 MB PDF) [file pcbi.1000564.s007.pdf]

# NS

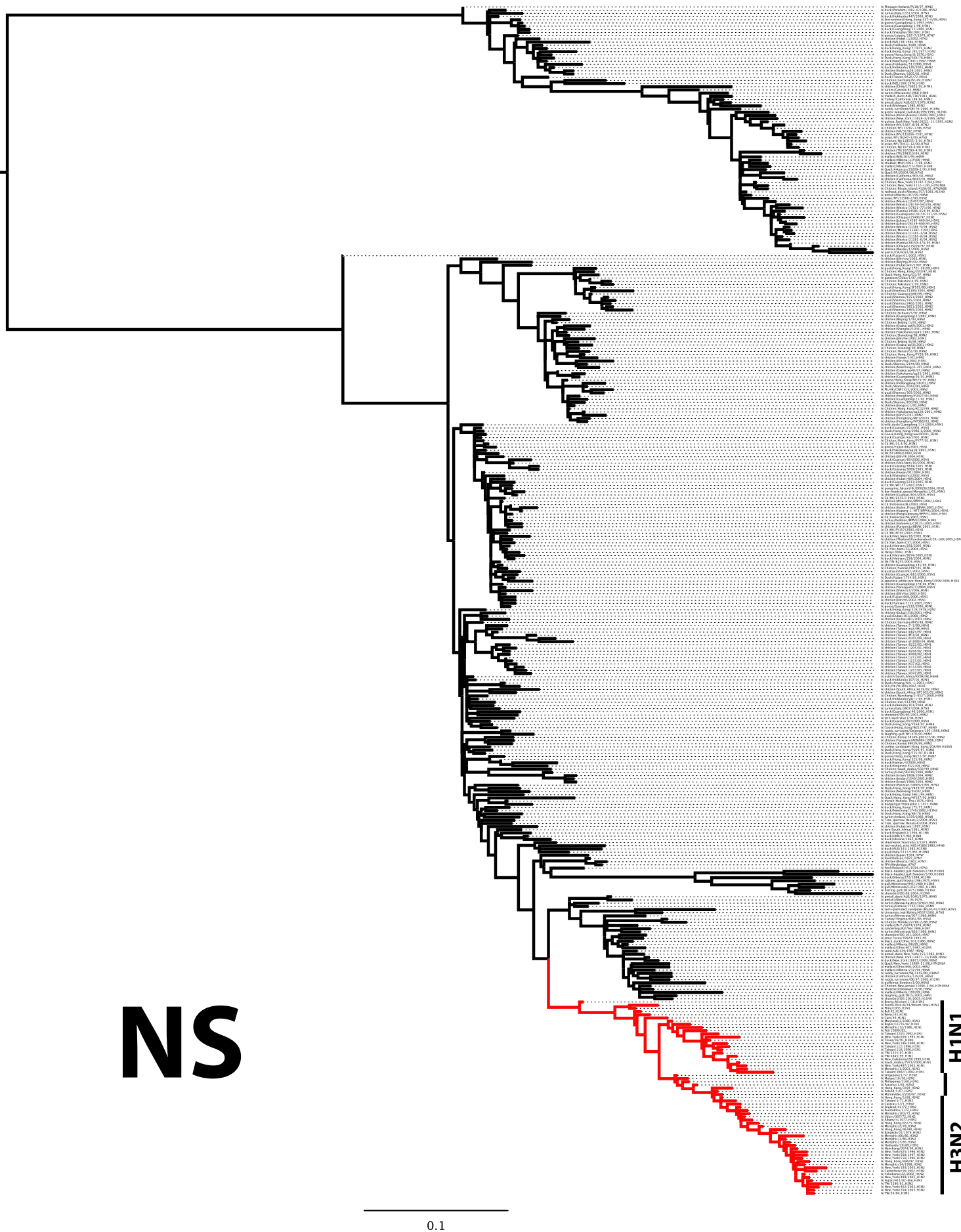

H3N2 H1N1  
H2N2

Supplement: Figure S7 — Phylogenetic tree of NS genomic segment. Avian section of the tree is in black, human in red. (2.94 MB PDF) [file pcbi.1000564.s008.pdf]

NP

0.1

H3N2 H2N2 H1N1

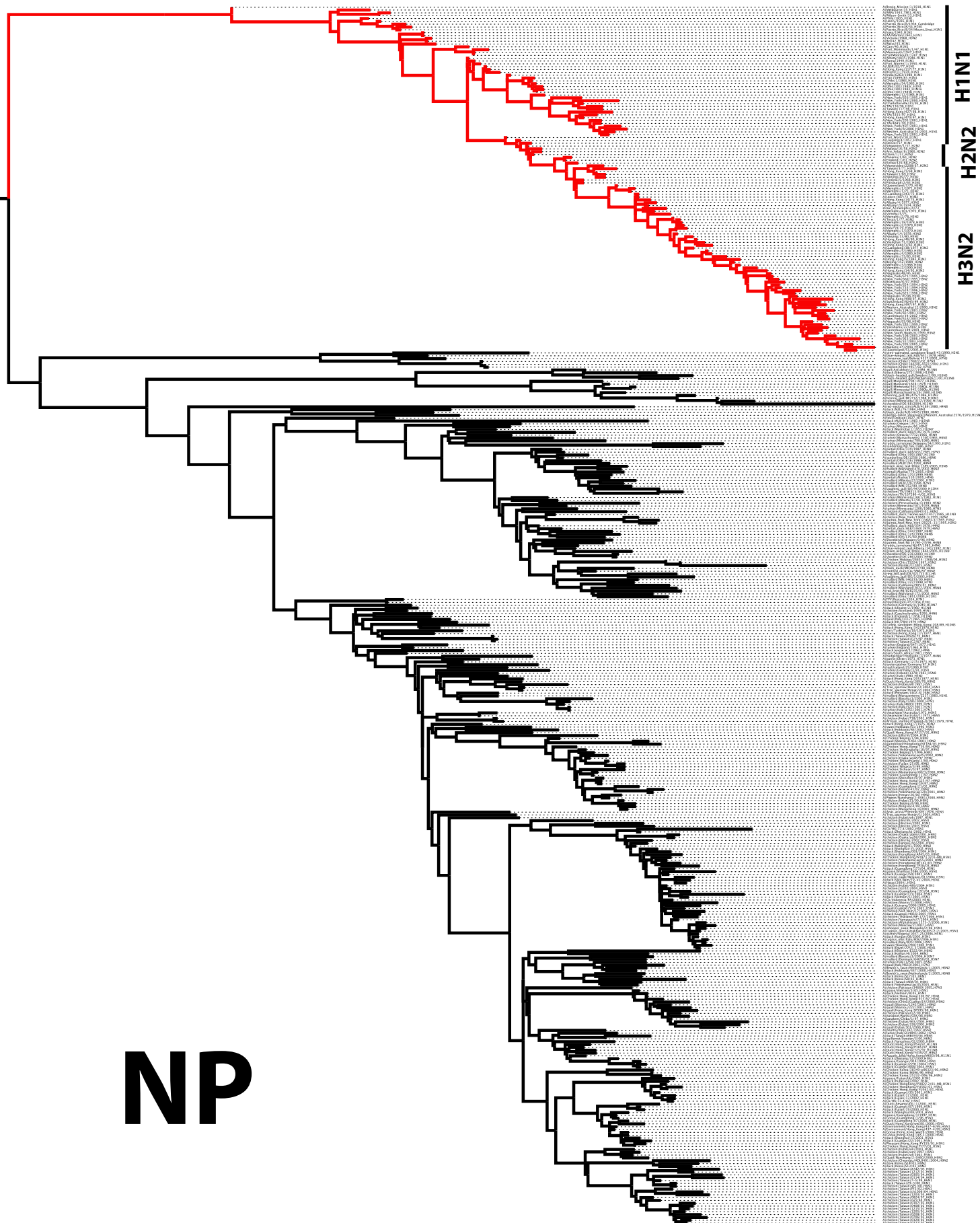

Supplement: Figure S8 — Phylogenetic tree of NP genomic segment. Avian section of the tree is in black, human in red. (3.28 MB PDF) [file pcbi.1000564.s009.pdf]

PA

0.1

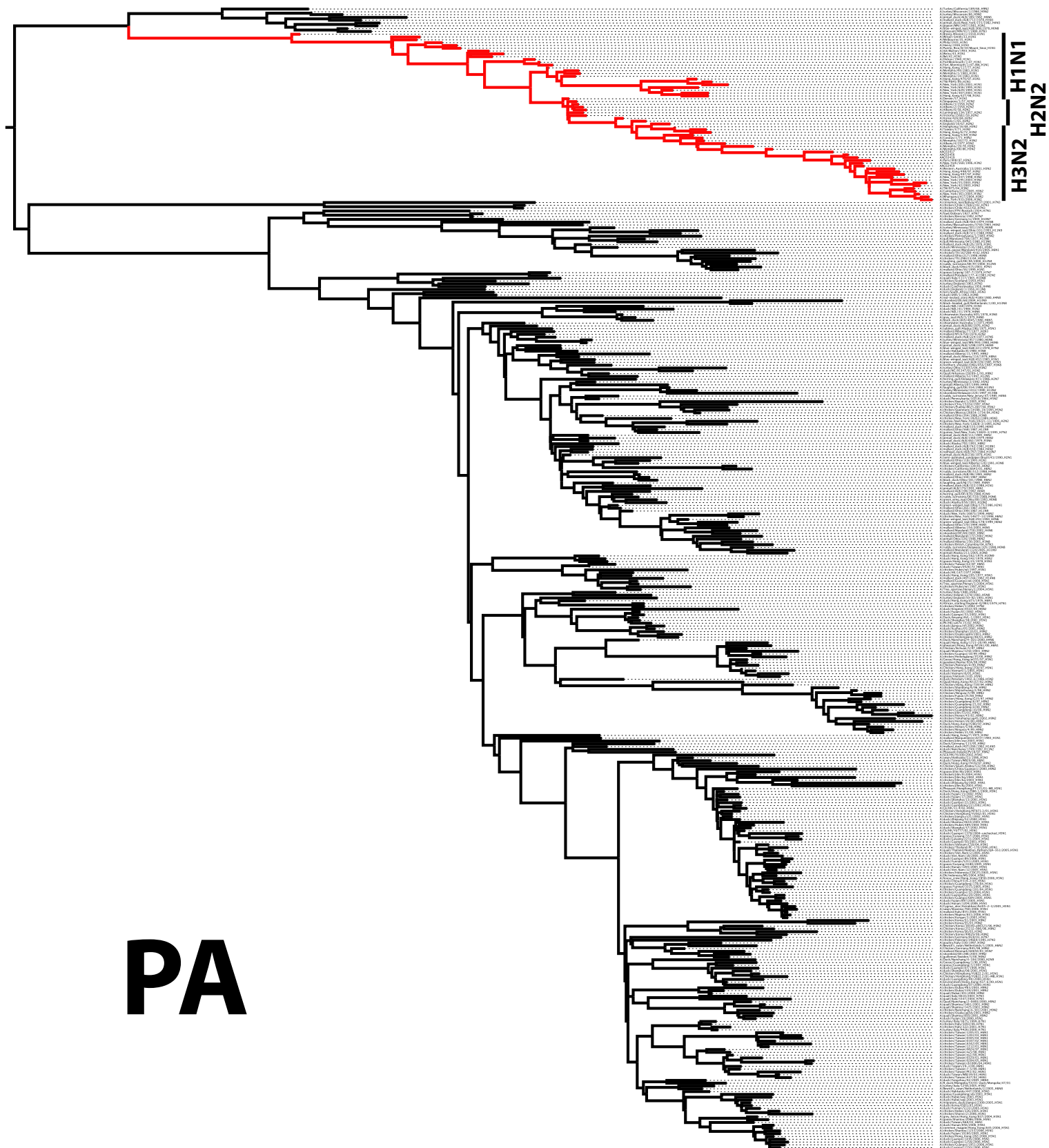

Supplement: Figure S9 — Phylogenetic tree of PA genomic segment. Avian section of the tree is in black, human in red. (3.25 MB PDF) [file pcbi.1000564.s010.pdf]

PB1

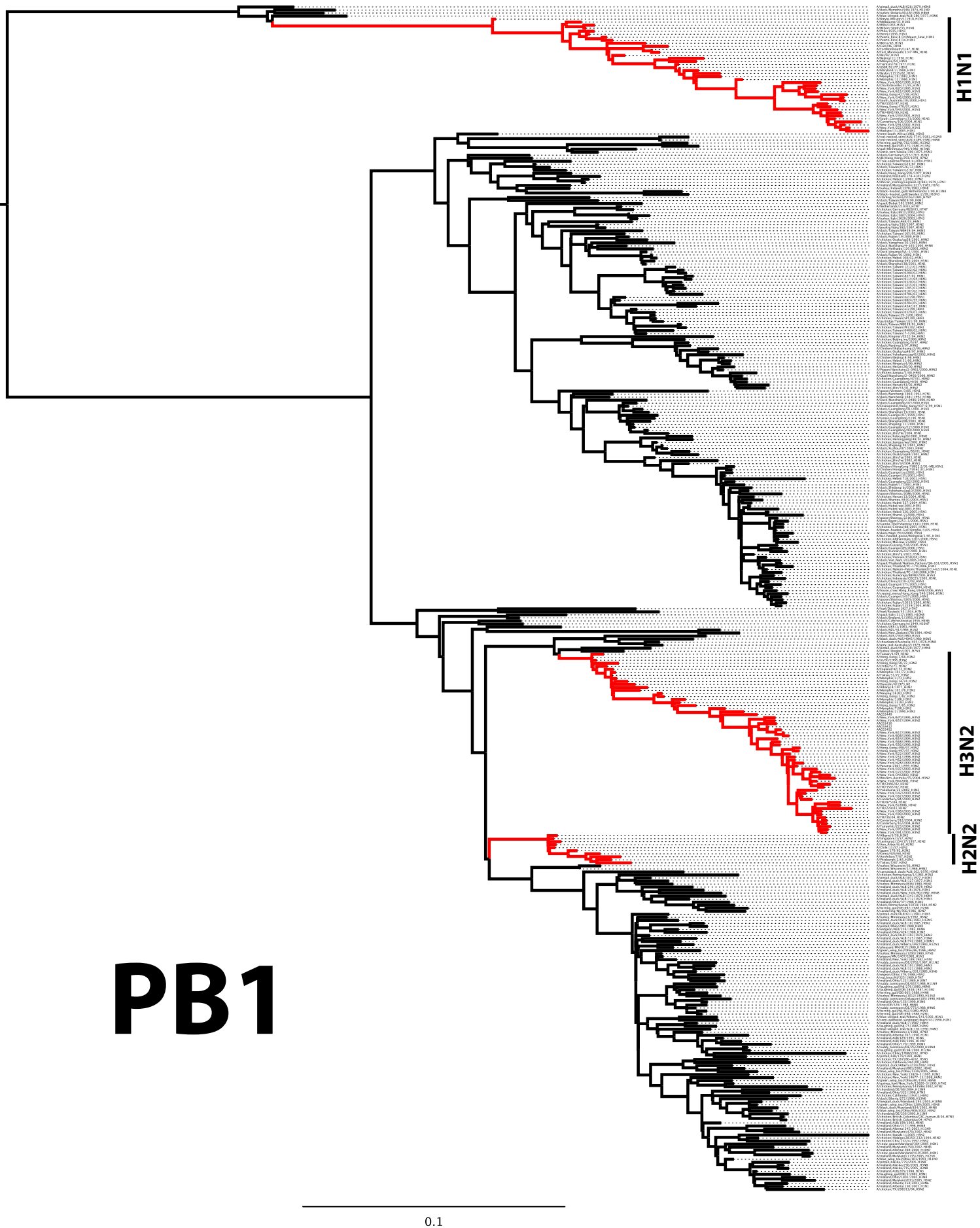

Supplement: Figure S10 — Phylogenetic tree of PB1 genomic segment. Avian section of the tree is in black, human in red. (3.03 MB PDF) [file pcbi.1000564.s011.pdf]

PB2

0.1

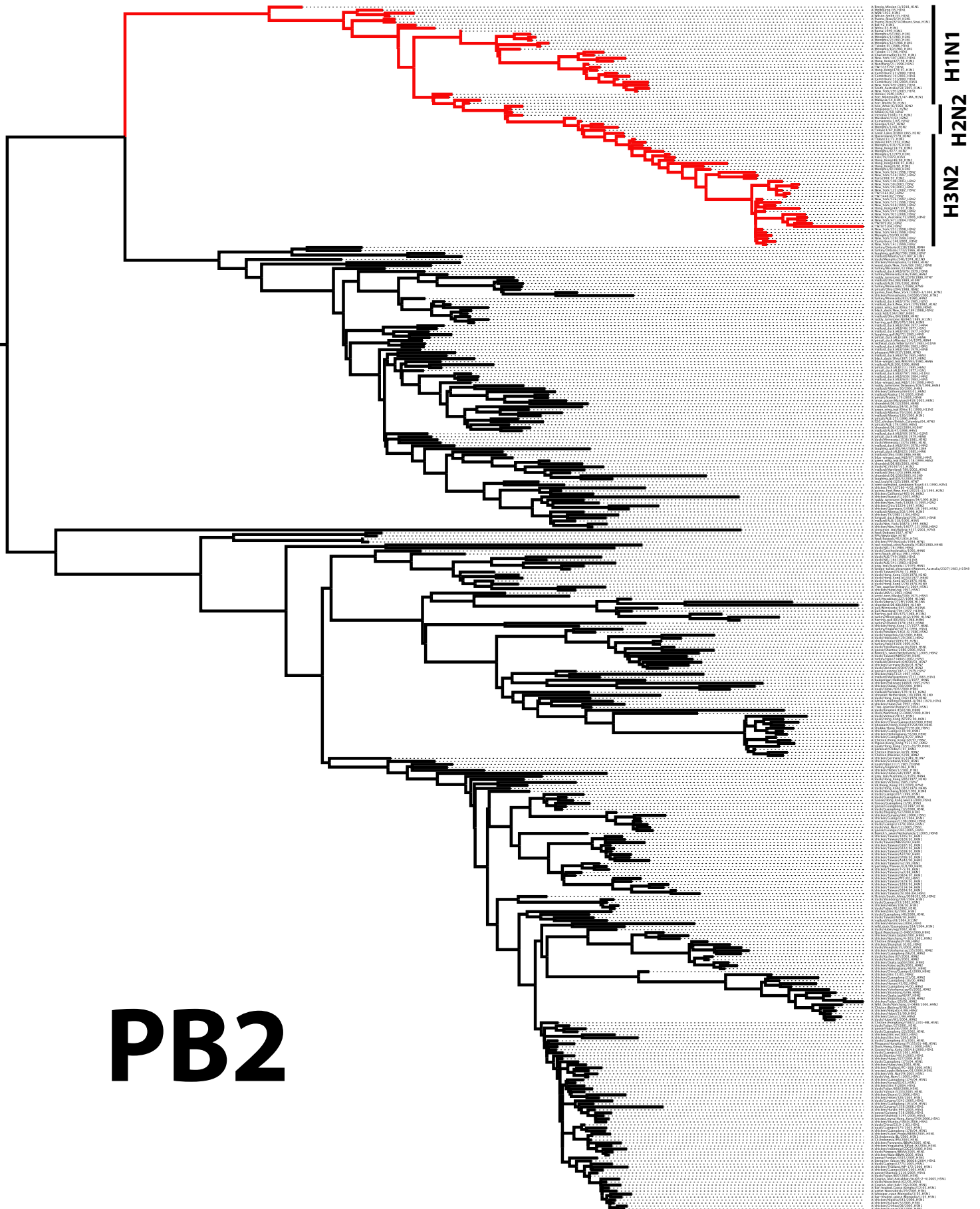

Supplement: Figure S11 — Phylogenetic tree of PB2 genomic segment. Avian section of the tree is in black, human in red. (3.21 MB PDF) [file pcbi.1000564.s012.pdf]
